# Differentiating Disease Flare From Infection: A Common Problem in Rheumatology. Do 18F-FDG PET/CT Scans and Novel Biomarkers Hold The Answer?

**DOI:** 10.1007/s11926-018-0779-4

**Published:** 2018-09-17

**Authors:** Elizabeth Mabey, Andrew Rutherford, James Galloway

**Affiliations:** 1 King’s College London, London, UK; 2 King’s College Hospital NHS Foundation Trust, London, UK; 3 Department of Inflammation Biology, King’s College London, New Hunt’s House, Great Maze Pond, London, SE1 1UL UK

**Keywords:** Fever, FUO, Rheumatology, Diagnostics, Iimaging, Biomarkers

## Abstract

**Purpose of review:**

Fever is common within rheumatology but it is often challenging to identify its source. To do so correctly is paramount in patients with an underlying inflammatory condition receiving immunosuppressive therapy. This review article looks at the available evidence and merits of both 18F-fluoro-deoxyglucose positron emission tomography/computed tomography (18F-FDG PET/CT) scans and new proposed biomarkers in determining the cause of fever within rheumatology.

**Recent findings:**

18F-FDG PET/CT scans are already an established tool in the detection and diagnosis of malignancy and are emerging for use in fever of unknown origin. More recently, they have been used to identify rheumatological causes of fever such as large vessel vasculitis and adult-onset Still’s disease. Within these conditions, biomarkers such as procalcitonin and presepsin may help to differentiate endogenous from exogenous pyrogens.

**Summary:**

18F-FDG PET/CT scanning shows promise in locating the source of pyrogens and may be superior to other conventional forms of imaging. As evidence and test availability increases, its use is likely to become commonplace in the diagnostic work-up of fever. Once a source is located, selected biomarkers may be used to confirm a cause.

## Introduction

Fever is an adaptive response to pyrogens that frequently presents rheumatologists with a challenge. In particular, the difficulty lies in determining the originating location of these pyrogens and whether they are endogenous or exogenous. The original definition of fever of unknown origin (FUO) from Petersdorf and Beeson is a febrile illness lasting for more than 3 weeks’ duration with a body temperature of >38.3°C on several occasions and no resulting diagnosis despite extensive investigations with at least 1 week of inpatient investigation [1]. In 1991, Durack and Street revised these criteria with two main changes [2]. Firstly, they shortened the duration of investigation to either 3 outpatient visits or 3 days of inpatient investigation, reflecting better access to advanced diagnostics in modern medicine. Secondly, they divided FUO into four distinct classes. These are classical FUO, neutropaenic FUO, nosocomial FUO and HIV-associated FUO. It is important to make the distinction, as the distribution of underlying aetiologies will vary between the groups, with more infections seen in the latter three categories compared with classical FUO.

No final diagnosis is reached in 7–53% of FUO patients [3]. Where a diagnosis is reached, the main causes can be divided into infection, malignancy and non-infectious inflammation. It is estimated that 20% cases worldwide are caused by a non-infectious inflammatory disorder, most commonly large vessel vasculitis and adult-onset Still’s disease (AOSD) [4].

When a febrile patient already has an established underlying inflammatory condition, identification between infection and acute flare is paramount to facilitate correct and safe management. To administer immunosuppressive treatment mistakenly in infection could be catastrophic. This distinction can be challenging, and has by and large been based upon clinical reasoning. However, recent developments in nuclear imaging and biomarkers may in future aid the clinician in making this distinction. This article will look at the utility of these developments within rheumatology, with the focus on the use of 18F-fluoro-deoxyglucose positron emission tomography/computed tomography (18F-FDG PET/CT) scans in identifying a pyrogen location and biomarkers such as procalcitonin for distinguishing the endogenous from the exogenous.

## 18F-FDG PET/CT Scanning

18F-FDG PET/CT scans are already an established tool in the detection and diagnosis of malignancy, and are emerging for use in fever of unknown origin (FUO) [5]. 18F-fluoro-deoxyglucose (18F-FDG) is injected into the bloodstream and enters cells via glucose transporter proteins. Areas of higher concentration can be visualised using PET/CT imaging. Active transporter proteins take up more 18F-FDG, for example when there is increased cell metabolism such as in malignant, infectious and inflammatory processes [6]. Thus, it differs from traditional CT and MRI scanning in that it looks at functional rather than anatomical processes. Functional images allow differentiation between active and residual inflammation with less nephrotoxicity and radiation than a contrast CT chest, abdomen and pelvis scan [6, 7, 8•].

A fundamental disadvantage of the 18F-FDG PET/CT scan is that, while often able to locate the source of the pyrogens, it is unable to distinguish between malignant, infectious or inflammatory processes. Instead, it may be used effectively to guide further investigation [6]. There are issues of cost and accessibility when compared to CT or MRI scanning, although some of the cost may be negated by reduced hospital admission length [6, 8•]. A further issue relates to the reliability of the results. False positive uptake of 18F-FDG has been shown to occur in macrophages of atherosclerotic plaques in the aorta and iliofemoral regions [9•]. Excretion of 18F-FDG by the kidneys may mask pelvic anomalies, resulting in false negative results [10]. In addition, 18F-FDG uptake may be affected by glucocorticoid or other immunosuppressant treatments commonly used within rheumatology [11]. One study showed glucocorticoid treatment of longer than 10 days duration to decrease uptake of 18F-FDG [9•].

Evidence for its utility in practice is limited, with no large-scale studies available. A recent meta-analysis of 18 studies suggested that 18F-FDG PET/CT scanning provided a 56% diagnostic contribution in patients with FUO [8•]. Of these scans, 9% resulted in false positives and 73% patients obtained a final diagnosis. The authors comment that most of the studies in the analysis were observational, with no control and much room for bias. Inevitably, more studies are needed before definitive answers are provided for its use in FUO.

### 18F-FDG PET/CT Scanning in Fever of Unknown Rheumatological Origin

There is a growing body of research focused on the diagnostic contribution of 18F-FDG PET/CT scanning in specific rheumatological conditions. This section looks at the two most common non-infectious causes of FUO: large vessel vasculitis and AOSD.

#### Large Vessel Vasculitis

Large vessel vasculitides such as giant cell arteritis (GCA) and Takayasu’s arteritis are common inflammatory causes of FUO. GCA presents with fever in a third of patients and Takayasu’s arteritis in 20% [12, 13]. 18F-FDG PET/CT scanning has been proposed to be superior to other forms of imaging in both forms [14]. This is because, early in the course of the disease, anatomical changes may be absent and thus may be missed by conventional imaging, such as CT and MRI scanning [15]. Furthermore, the gold standard diagnostic tool for GCA of a superficial temporal biopsy has high false negative rates of 15–40% due to skip lesions [16]. A systematic review showed sensitivities and specificities of 18F-FDG PET/CT scanning in GCA of 90% and 98% and in Takayasu’s of 87% and 73%, respectively [17]. Another study looked at 18F-FDG PET/CT scanning in patients with large vessel vasculitis compared to those with hyperlipidaemia and vasculitis mimics, and found that 18F-FDG PET/CT scanning could differentiate between these with a sensitivity of 85% and specificity of 83% [18•]. Despite being unable to view the temporal artery, the characteristic distribution of uptake of 18F-FDG in the PET/CT scan may be used to distinguish between these forms of vasculitis. In GCA, 18F-FDG accumulates in the aorta, subclavian, carotid and iliac arteries, whereas in Takayasu’s arteritis, it is more centrally located in the aorta and its main branches in the thoracic region [19].

#### Adult-Onset Still’s Disease

AOSD has been referred to as the archetypal febrile autoinflammatory illness [20]. It typically causes a diurnal fever, highest in the evening accompanied by a salmon pink rash and arthralgia. Currently, the diagnosis is largely clinical due to low prevalence, vague symptoms and the absence of any diagnostic test [21]. Data regarding utility of 18F-FDG PET/CT scanning are limited, but 18F-FDG has been shown to accumulate in bone marrow, spleen, lymph nodes and joints in the condition, and thus may be helpful when combined with clinical features [22]. One limitation is an inability to distinguish AOSD from lymphoma. However, it may be used to guide an appropriate site for biopsy, thus facilitating the correct diagnosis [22].

## Use of Biomarkers to Differentiate Endogenous and Exogenous Pyrogens

While 18F-FDG PET/CT scanning is useful for determining a location for pyrogens, the differentiation between infection and acute flare of an autoimmune condition remains a challenge for the rheumatologist. Standard biomarkers for infection, such as a white cell count (WCC), erythrocyte sedimentation rate (ESR) and C-reactive protein (CRP), may be elevated in both conditions. An exception to this is in systemic lupus erythematosus (SLE), where CRP does not typically rise in acute flare but does in concurrent infections rendering it a useful biomarker for infection [23]. Microbiological investigations may also have limitations such as temporal delays and low accuracy rates [24].

### Procalcitonin

To combat these problems, a hunt for specific biomarkers to aid differentiation between the two has ensued. One proposed biomarker is procalcitonin. This is a precursor of calcitonin which rises substantially in bacterial infection but not normally in non-infectious inflammatory diseases [25]. However, a moderate rise has been seen in active AOSD and vasculitis in the absence of infection [26]. A meta-analysis of suspected septic arthritis and osteomyelitis revealed that procalcitonin had a sensitivity of 67% and specificity of 90%, but, at lower serum concentrations of procalcitonin, the sensitivity rose to 87% [27]. It has been shown to differentiate bacterial infection from acute flare in rheumatoid arthritis, with superiority over conventional biomarkers such as CRP, ESR and WCC [28]. Conversely, as illustrated earlier, CRP is a sensitive marker for bacterial infection in SLE and has been shown to be superior to procalcitonin as a biomarker for this condition [29]. Concomitant glucocorticoid use does not seem to affect procalcitonin levels, but evidence for other immunosuppressive therapies is lacking [30].

### Presepsin

A second proposed biomarker is presepsin. This is a soluble CD14 subtype which is thought to be released following cellular phagocytosis in bacterial infections, and has been shown to be useful in management of sepsis [31, 32]. Evidence for its use in rheumatology is limited, but one study shows promise and superiority of presepsin over procalcitonin in rheumatoid arthritis patients with low-grade bacterial infections with a sensitivity and specificity of 92.3% and 77.8%, respectively [33•]. Another study suggests that baseline presepsin levels in rheumatoid arthritis patients without infection is higher than in healthy controls [34]. However, careful use of cut-off values helped to distinguish between these rheumatoid arthritis patients with and without infection. In addition, their proposed cut-off levels were unaffected by the use of glucocorticosteroids and methotrexate therapy.

### Combined Biomarkers

Finally, a number of studies have considered the utility of combined biomarkers, such as CRP with interleukin-6 (Il-6) in periprosthetic joint pathology showing superiority over other biomarkers (including procalcitonin) when distinguishing between aseptic joint loosening and infection [35•, 36]. Studies have not yet compared CRP and Il-6 levels in inflammatory and infectious processes within rheumatological conditions. Moreover, CRP and Il-6 may both be raised in an acute flare of such conditions. However, as with presepsin, further research may reveal a useful cut-off value to help differentiate the two.

In summary, these novel biomarkers show promise in the differentiation between endogenous and exogenous pyrogens, but are not yet in routine clinical practice. With further research, they may aid diagnosis in certain patient groups.

## Conclusion

Fever can be a very challenging presentation due to its variety of different aetiologies. Correct and rapid identification is important and especially so in rheumatology where patients may be on immunosuppressant therapy. Recent developments have meant that we may be closer to pinpointing a source of pyrogens. 18F-FDG PET/CT scanning can help to locate the pyrogens and, with further research, its use may become more routine in the diagnostic work-up of fever. Consequently, it may help to diagnose common causes of fever often missed, such as large vessel vasculitides and AOSD. Concurrent use of biomarkers may further facilitate diagnosis in the differentiation between endogenous and exogenous pyrogens. In future, it may be used by rheumatologists for patients with underlying inflammatory pathology to aid differentiation between infectious and acute inflammatory flares.
